# Wire Electrochemical Etching of Superhydrophobic Nickel Surfaces with Enhanced Corrosion Protection

**DOI:** 10.3390/ma16237472

**Published:** 2023-12-01

**Authors:** Binghan Wu, Defeng Yan, Junyi Lin, Jinlong Song

**Affiliations:** 1 State Key Laboratory of High-Performance Precision Manufacturing, Dalian University of Technology, Dalian 116024, China; wubinghan@mail.dlut.edu.cn (B.W.); yandefeng1994@163.com (D.Y.); linjy1372@163.com (J.L.); 2 Key Laboratory for Micro/Nano Technology and System of Liaoning Province, Dalian University of Technology, Dalian 116024, China

**Keywords:** superhydrophobic, corrosion protection, wire electrochemical etching, nickel surface

## Abstract

Superhydrophobic nickel surfaces have significant advantages in the field of corrosion protection compared with traditional nickel corrosion protection methods which need a toxic chemical corrosion inhibitor. Electrochemical etching, an ideal method for fabricating superhydrophobic nickel surfaces, was also limited by low current density, resulting in low processing efficiency. To overcome this limitation, we proposed a new method to fabricate a superhydrophobic nickel surface using a wire electrochemical etching method. The wire electrochemical etching method accomplished the etching process by sweeping a controlled wire cathode across the surface of the anode nickel plate in an environmentally friendly neutral electrolyte, NaCl. The superhydrophobic nickel sample with a contact angle of 153° and a rolling angle of 10° could be fabricated by wire electrochemical etching and modification. Additionally, the optimal parameters of the wire electrochemical etching and the principle of superhydrophobic surface formation had also been systematically investigated, respectively. Moreover, the superhydrophobic nickel surface had self-cleaning performance, antifouling performance, corrosion protection, and abrasion resistance. Wire electrochemical etching improves the current density of processing, which means that this method improves the processing efficiency for fabricating a superhydrophobic nickel surface. This work is expected to enrich the theory and technology for fabricating superhydrophobic nickel surfaces to improve the corrosion protection of nickel.

## 1. Introduction

Nickel, as an industrial metal, has extremely important applications in many engineering fields, such as airplanes, radars, and batteries, due to its excellent ductility, magnetism, and mechanical properties [1,2,3,4]. In addition, nickel itself is not easily corroded and is widely used as a protective layer on device surfaces [5,6]. Although nickel can be used as a protective metal, its corrosion protection is limited. If an object made of nickel or containing a part with nickel as the outer protective layer is exposed to the natural environment for a long time, its surface often comes into contact with water, which can still cause surface corrosion and cause parts to fail [7]. Therefore, methods of improving the corrosion protection of nickel surfaces in natural environments have attracted widespread attention from researchers.

Initially, people used paint or chemical corrosion inhibitors to protect metal surfaces from corrosion [8,9]. However, these methods have some problems, such as the corrosion-protective paint often emitting irritating and toxic odors, as well as the chemical corrosion inhibitor’s high cost [10,11]. Many attempts have been made to improve the corrosion protection of the nickel surface, but it was still difficult to realize a low-cost, non-toxic corrosion-protective method until 1999 [12]. In that year, Nakajima discovered that a superhydrophobic surface made it difficult for water to penetrate due to an air film layer on the surface. This revelation offered the potential to address the limitations of traditional metal corrosion-protective technology [13,14,15]. Researchers have developed various methods to fabricate a superhydrophobic surface on the metal substrate, which can achieve self-cleaning, corrosion protection, anti-icing, droplet manipulation, water collection, and other functionalities [16,17,18,19]. However, compared with other metal materials, relatively few studies have focused on fabricating a superhydrophobic nickel surface, with the main fabrication methods being laser processing [20,21,22], electro-deposition [23,24,25], chemical etching [26,27,28], and so on. However, these methods have some limitations: the laser fabrication demanded costly equipment and had low processing efficiency; the electro-deposition formed superhydrophobic nickel coatings with poor mechanical strength; and the chemical etching required strong oxidants to obtain desired structures, which was environmentally unfriendly. In 2020, Ma proposed a method for fabricating a superhydrophobic nickel surface using electrochemical etching [29]. This method used a non-toxic neutral electrolyte of NaNO_3_ and NaCl to fabricate the nickel surface, and the surface was modified with low surface energy to achieve superhydrophobicity. Although this method can fabricate superhydrophobic nickel in a facile and green method, its cathode was a plate cathode, which meant that the current density was low, resulting in low fabrication efficiency [30]. Therefore, to meet the practical fabrication requirement, a facile, environmentally friendly, high current density electrochemical etching method needs to be proposed to achieve efficient fabrication of a superhydrophobic nickel surface.

To overcome the limitations of plate electrochemical etching, we proposed a wire electrochemical etching method that can be facile, environmentally friendly, and highly efficient to fabricate a superhydrophobic nickel surface. We first demonstrated that the wire electrochemical etching had higher potential and current density than the plate electrochemical etching method using COMSOL Multiphysics 5.6 software. To investigate the optimal parameters of wire electrochemical etching, we conducted a single factor experiment to determine the optimal fabrication parameters for three factors: feed rate, electrolyte concentration, and voltage. We obtained the superhydrophobic nickel samples with a contact angle of 153° and a rolling angle of 10° and analyzed the reasons for the generation of superhydrophobic surfaces. As a surface material, the superhydrophobic nickel surface also had better performance in self-cleaning, antifouling, corrosion protection, and abrasion resistance. This technology provided a new method to improve the corrosion protection of nickel material.

## 2. Experimental

### 2.1. Materials

Nickel plates (N4 #, size of 10 mm × 10 mm × 2 mm, purity 99.99%) were purchased from Rundle Metals Co., Ltd. (Yantai, China). Brass wire (diameter of 100 μm) was purchased from Yue-blue Precision Hardware Co., Ltd. (Dongguan, China). Sandpapers (1200 # and 2000 #) were purchased from Mercola Co., Ltd. (Shanghai, China). Fluoroalkylsilane [FAS, C_8_F_13_H_4_Si(OCH_2_CH_3_)_3_] was purchased from Degussa (Frankfurt, Germany). Analytical grade NaCl and ethanol were purchased from Bono Chemical Reagents Co., Ltd. (Dalian, China). The HCl used to prepare the pH = 1 solution and the NaOH used to prepare the pH = 14 solution were purchased from Bono Chemical Reagents Co., Ltd. (Dalian, China).

### 2.2. Fabrication of Sample

**Figure 1:**
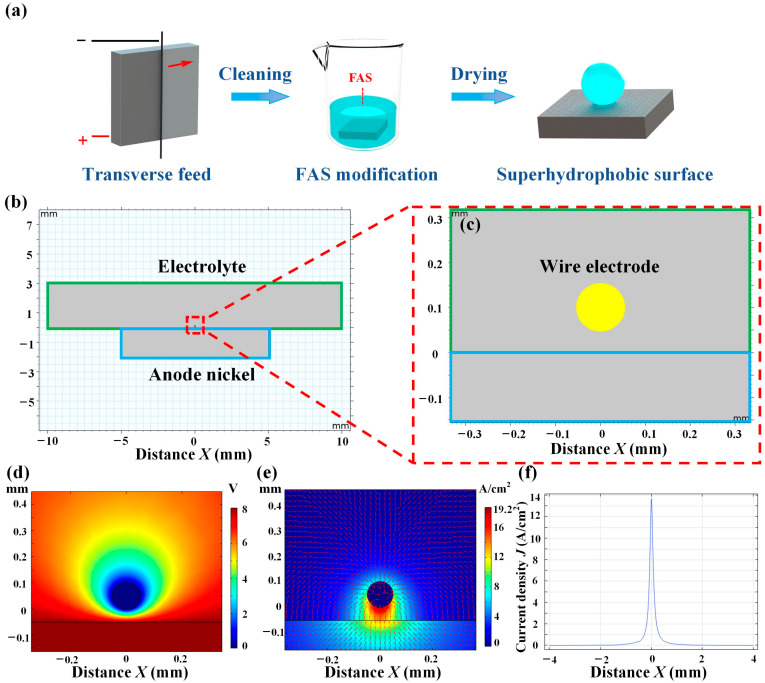
Wire electrochemical etching process and simulation. (**a**) Flow chart of superhydrophobic nickel sample for wire electrochemical etching. (**b**) Model for wire electrochemical etching. (**c**) Local magnification for wire electrochemical etching. (**d**) Voltage for wire electrochemical etching. (**e**) Current density for wire electrochemical etching. (**f**) Current density data on the surface of anode nickel plate.

The fabrication processes of electrochemically etching superhydrophobic nickel samples via the wire cathode are shown in Figure 1a. The roughness of the entire bare nickel surface was 0.96 μm, and then it was polished with the 1200 # and 2000 # sandpaper; the roughness of the polished surface reached up to 0.15 μm. Then, the polished sample was etched via wire electrochemical etching with 30 μm/s feed rate, 30 g/L NaCl electrolyte concentrations, and 8 V voltage. Next, the etched sample was cleaned in the ultrasonic cleaner (LT-05C, Longbiao Electric Co., Ltd., Jinan, China) and was immersed into the FAS-ethanol solution at a concentration of 1 wt% for one hour. Finally, the superhydrophobic nickel sample was obtained after drying in an oven at 70 °C for 20 min [31].

### 2.3. Characterization

The surface microstructures of the samples were observed using a scanning electron microscope (SEM, JSM-6360LV, Tokyo, Japan), which was equipped with energy-dispersive spectroscopy (EDS, JSM-6360LV, Tokyo, Japan). The crystal structure and phase analysis of the samples were determined by an X-ray diffractometer (XRD, Empyrean, Alemlo, Netherlands). The XRD diffraction angles spanned from 20° to 100°. The surface roughness of the samples was measured using a 3D surface profilometer (Zygo, NewView9000, Middletown, CT, USA). The contact angle (CA) and rolling angle (RA) were measured using an optical contact angle meter (Solon, SL200KS, Boston, MA USA) by dropping a 5 μL water droplet on the sample surfaces at room temperature. We used the average of five measurements for data processing.

### 2.4. Electrochemical Impedance Spectroscopy Tests

The electrochemical impedance spectroscopy tests were used to quantitatively characterize the corrosion protection of the superhydrophobic nickel plate and the bare nickel plate using an electrochemical workstation (Chenhua 760e, Shanghai, China). Two nickel plates with the size of 10 mm × 10 mm × 2 mm were used in the electrochemical impedance spectroscopy test. The electrolytic cell of the electrochemical workstation is a typical three-electrode system. The working electrode was the nickel plate. The auxiliary electrode was a graphite rod electrode, and the reference electrode was a saturated silver chloride electrode. The test solution is 3.5 wt% of NaCl solution. The high frequency was 10,000 Hz and the low frequency was 0.01 Hz. The amplitude was 5 mV.

## 3. Results

The traditional electrochemical etching method for constructing microstructures on metal substrates typically used a plate cathode [29]. However, this method had limitations, such as low current density, long processing time, and low efficiency when fabricating microstructures for superhydrophobic surfaces [30]. To address these issues, we proposed a wire electrochemical etching method. In our work, we utilized the COMSOL Multiphysics 5.6 software to build the models presented in Figure 1b,c and Figure S1a,b. The process of using both the wire cathode and plate cathode was then simulated. The parameters used in the simulation were detailed in Table 1.

During the electrochemical etching process, micro- and nanostructures were formed on the surface of the anode nickel plate via etching. This required a high current density in the surface of the anode. To achieve this, the gap between the anode and cathode needed to be relatively small while still allowing the flow of electrolytes. Furthermore, the processing area between electrodes should be as small as possible. 

Simulation results showed that in the plate electrochemical etching, the voltage was sparse, and the current density was low and concentrated at the two end point locations of the anode nickel surface, as shown in Figure S1c,d. In contrast, in the wire electrochemical etching, the voltage was intensive, and the current density was high and concentrated on the surface of the anode nickel plate, as shown in Figure 1d,e. To observe the current density on the anode nickel plate in detail, the current density distribution on the surface of the anode nickel plate under the application of the same voltage was shown in Figure S1e,f. When using a plate cathode, the maximum current density on the surface of the anode nickel plate was only 0.1 A/cm^2^. However, when using a wire cathode, the maximum current density reached up to 14 A/cm^2^. Wire electrochemical etching reduced the processing area of the cathode and reduced the gap between the anode and cathode while still ensuring a continuous flow of electrolytes. Consequently, the current density was much higher than that of the plate cathode process. Therefore, the use of a wire cathode to fabricate nickel superhydrophobic surfaces greatly improved efficiency.

The phenomenon of electrolytic reactions induced by the current through electrodes was revealed by Faraday’s first law, which stated that the amount of substance reacting electrochemically at the anode and cathode is directly proportional to the amount of electricity passing through the circuit. Additionally, the amount of electricity is proportional to the current in the circuit and the processing time. Faraday’s second law reveals that in an electrolytic reaction, the substance dissolved at the anode and precipitated at the cathode have the same amount of substance. This led to the mass *M* of dissolved metal at the anode:

(1) M=kQ=kIt

where *M* is the amount of dissolved or precipitated material on the electrode (g), *k* is the mass chemical equivalent of the electrolyzed material [g/(A·s)], *Q* is the total charge through the interface of the anode and cathode (A·s), *I* is the processing current (A), and *t* is the processing time (s) [32]. In the actual electrochemical etching process, the volume change after the electrochemical etching led to micrometer-scale pits [33]. The electrochemical etching volume *V* according to the volume equation is:

(2) $$V=\frac{M}{\rho }=\frac{kIt}{\rho }=\omega It$$

where *V* is the volume of the substance dissolved at the anode (cm^3^); *ρ* is the density of the nickel (g/cm^3^); and *ω* is the volumetric electrochemical equivalent of the nickel [cm^3^/(A·s)] [34].

In the electrochemical etching process, the actual amount of the anode metal dissolved differs from the theoretical dissolved amount. Therefore, the current machining efficiency *η* is introduced [35]. Then, the formula is modified as:

(3) $$M_{e}=\eta kQ=\eta kIt$$

(4) $$V_{e}=\eta \frac{M}{\rho }=\eta \frac{kIt}{\rho }=\eta \omega It$$

During the wire electrochemical etching, the actual dissolution velocity of anode nickel plate in the direction of cathode feed rate *v*, for example, with an electrolytic area of *S*, is:

(5) $$v_{e}=\frac{\eta V}{St}=\frac{\eta \omega It}{St}=\eta \omega i$$

where *V* is the volume of the substance dissolved at the anode (cm^3^); *ω* is the volumetric electrochemical equivalent of the nickel [cm^3^/(A·s)]; and *i* is the current density (A/cm^2^) [36]. The resulting electrochemical corrosion pits are the basis for further preparation of superhydrophobic surfaces.

**Figure 2:**
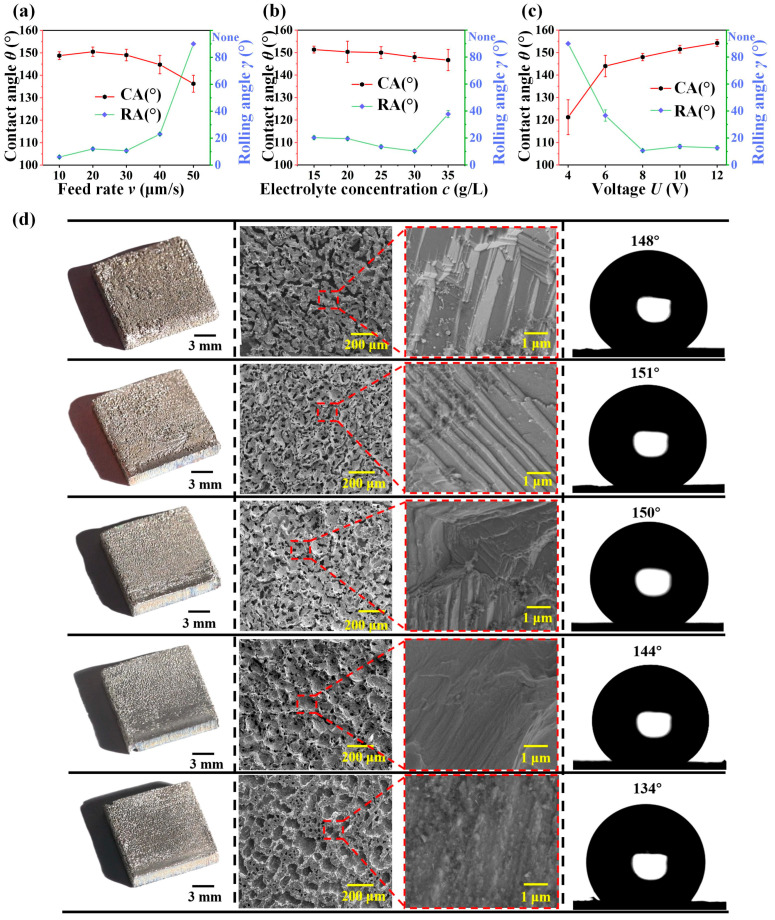
Effect of parameters on fabricating superhydrophobic surface. (**a**) Trend plot of the effect of feed rate on fabricating effect at 8 V and 30 g/L electrolyte concentration. (**b**) Trend plot of the effect of electrolyte concentration on fabricating effect at 30 μm/s feed rate and 8 V. (**c**) Trend plot of the effect of voltage on fabricating effect at 30 μm/s feed rate and 30 g/L electrolyte concentration. (**d**) Macroscopic, SEM, and contact angle measurements after etching at 10 μm/s, 20 μm/s, 30 μm/s, 40 μm/s, and 50 μm/s feed rate at 30 g/L electrolyte concentration and 8 V voltage.

Figure 2a–c showed the optimal superhydrophobic surface formed by the wire electrochemical etching through the comparison of different fabrication parameters. In this work, the effects on wettability were explored in terms of wire cathode feed rate, electrolyte concentration, and voltage. Through the preliminary pre-experiment, a range of parameters for fabricating superhydrophobic nickel samples were obtained, with a feed rate of roughly 10–50 μm/s, electrolyte concentration of 15–35 g/L, and a voltage of 6–10 V. Firstly, the electrolyte concentration of 30 g/L and the voltage of 8 V were selected to explore the effect of fabricating surface at different feed rates. We used the SEM to observe microstructures under different parameters. Figure 2d showed the surface effects obtained at various wire cathode feed rates, ranging from 10 μm/s to 50 μm/s. In each row, the feed rates increased from top to bottom. At 10 μm/s, the corrosion micro-pits can be clearly seen. With the increase of the feed rate, the corrosion traces on the surface of the nickel plate gradually became lighter and more uniform. This was due to the fact that at low rates, the anode nickel plate was etched for a long period of time and a richer micro-nanostructure was formed on the surface, resulting in better superhydrophobicity. When the feed rate exceeded 30 μm/s, the rolling angle increased rapidly with a further increase in feed rate, and there was no superhydrophobicity on the etched surface. Comparing the 10–30 μm/s process, a lower rolling angle can be obtained in the 10 μm/s process. However, in terms of efficiency, using a feed rate of 30 μm/s not only met the requirements for fabricating superhydrophobic nickel samples, but also greatly improved the efficiency of the fabrication. It was also possible to fabricate superhydrophobic surfaces with a contact angle of 153° and a rolling angle of 10°. Thus, the feed rate of 30 μm/s was selected.

Similarly, the feed rate of 30 μm/s and the voltage of 8 V were selected to explore the effect of fabricating surface at different electrolyte concentrations. As shown in Figure 2b, the contact angle remained relatively consistent across different electrolyte concentrations, which was greater than 150°. However, the rolling angle decreased greatly with the increase of electrolyte concentration at 15–30 g/L, indicating the increase of superhydrophobicity, and the superhydrophobicity decreased after exceeding 30 g/L electrolyte concentration. As a result, the electrolyte concentration of 30 g/L was selected. To explore the effect of voltage on wettability, 30 μm/s feed rate and 30 g/L electrolyte concentration were selected, as shown in Figure 2c, and it was summarized that better superhydrophobicity could be obtained when the voltage was 8 V. In summary, the best parameters were the feed rate of 30 μm/s, the electrolyte concentration of 30 g/L, and the voltage of 8 V. The variation in microstructure with electrolyte concentration and voltage surface change are shown in Figures S2 and S3.

**Figure 3:**
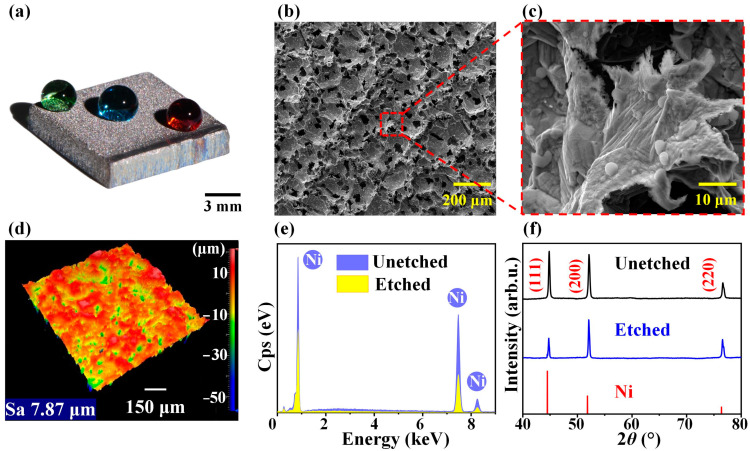
The characterization after fabricating. (**a**) Macroscopic image after fabrication. (**b**) SEM image of the surface after fabrication. (**c**) Local SEM magnification (**d**) Zygo image of the surface after fabrication. (**e**) EDS spectrum before and after fabrication. (**f**) XRD spectra before and after fabrication.

As shown in Figure 3a,b, the microstructure of the nickel plate surface was corrosion pits, which are necessary to form a superhydrophobic surface [37,38,39]. Upon further magnification of the microstructure, we observed that the stepped laminar structure existed in the edges of corrosion pits, which was due to grain boundary corrosion and dislocation corrosion that occurred during the electrochemical etching process and further expanded to produce corrosion pits, as shown in Figure 3c [40,41]. To investigate the size of the microstructures, we used Zygo to measure the surface roughness, as shown in Figure 3d. The average roughness of the entire surface after etching was 7.87 μm, and the corrosion pits were analyzed to explore their influence on superhydrophobicity. To investigate the variation in the element types before and after electrochemical etching, we used the EDS to analyze the surface, as shown in Figure 3e. The results showed that there was no variation in the element types before and after wire electrochemical etching. To investigate the variation in the structure and the composition of the crystalline material before and after electrochemical etching, we used the XRD to analyze the surface, as shown in Figure 3f. The results showed no variation before and after electrochemical etching compared with the bare nickel plate (PDF Card #04-0850).

**Figure 4:**
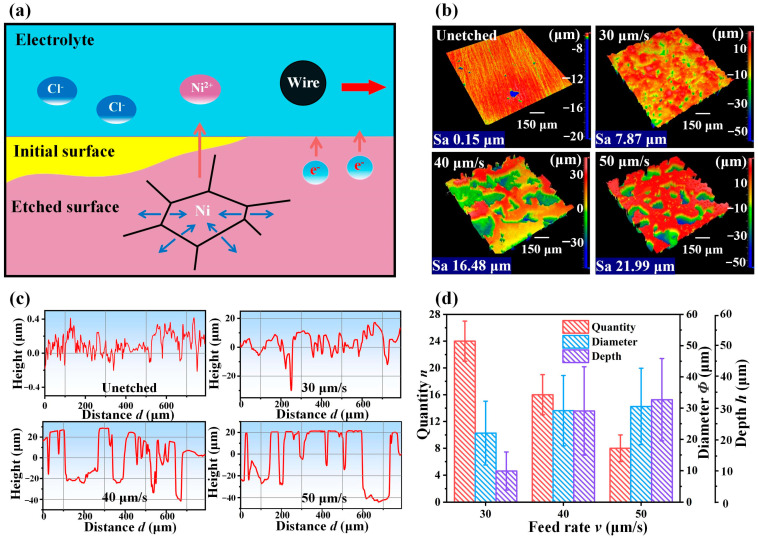
Fabrication schematic and analysis of the principle of superhydrophobicity. (**a**) Schematic diagram of the principle of electrochemical etching process. (**b**) Image of the surface at a feed rate of 30–50 μm/s at 8 V and 30 g/L electrolyte concentration. (**c**) Plot of the measured surface profile data at a feed rate of 30–50 μm/s at 8 V and 30 g/L electrolyte concentration. (**d**) Statistical diagram of the size and quantity of corrosion pits.

The processing principle was further analyzed and the reason for the variation in wettability was explored by measuring the complexity of the microstructure, as shown in Figure 4. The presence of numerous grain boundaries and dislocations in nickel resulted in preferential corrosion at these locations during wire electrochemical etching processing, as shown in Figure 4a. As a result, when the wire cathode swept across the surface of the nickel anode plate, the stepped laminar structure initially formed at the grain boundaries, and corrosion gradually extended towards the center of the grains, resulting in the formation of corrosion pits. To investigate the influence of various processing parameters on the complexity of the surface microstructure and analyze their effect on wettability, we conducted measurements of the surface microstructure after etching using Zygo (NewView9000, Middletown, CT, USA) at different feed rates of 30 g/L electrolyte concentration and 8 V voltage, as shown in Figure 4b. First, the surface roughness of the polished and unetched nickel plate was compared with that of the nickel plate after etching at 30 μm/s, 40 μm/s, and 50 μm/s feed rates. On the unetched surface, it appeared smooth, with a roughness of only 0.15 μm over the entire surface. The roughness was slightly reduced when the feed speed was low, measuring approximately 7.87 μm. However, as the speed increased, the roughness became noticeably higher. The difference in roughness indicated that the distance between the peaks and valleys of two adjacent corrosion pits on the surface of the etched surface was smaller in the low feed rate, and the value increased as the feed rate increased.

The surface morphology and characteristics of corrosion pits, including the quantity, depth, and diameter, were further analyzed by Zygo, as shown in Figure 4c. When the wire cathode was etching at a feed rate of 30 μm/s, the surface was entirely corroded, as shown in Figure 4c. It formed intricate corrosion pits, and the diameter and depth of the corrosion pits were relatively small. When the feed rate was increased to 40 μm/s, it can be seen from the surface height data that some areas were obviously not corroded. When the feed rate was further increased to 50 μm/s, most of the areas were not corroded. The quantity, depth, and diameter of the surface microstructures at different feed rates were quantified, as shown in Figure 4d. When the feed rate was increased, the number of pits decreased; both the depth and diameter of the pits increased, which meant that the complexity of the microstructure became increasingly simple. As a result, the contact angle of the etched surface showed a significant decrease and the rolling angle increased dramatically when the feed rate was faster than 30 μm/s. The surface in this case could not achieve a superhydrophobic effect. In summary, when the feed rate exceeded 30 μm/s, the originally rich microstructure became simple, and thus it was difficult to meet the structural requirements of superhydrophobicity, and superhydrophobicity was lost.

**Figure 5:**
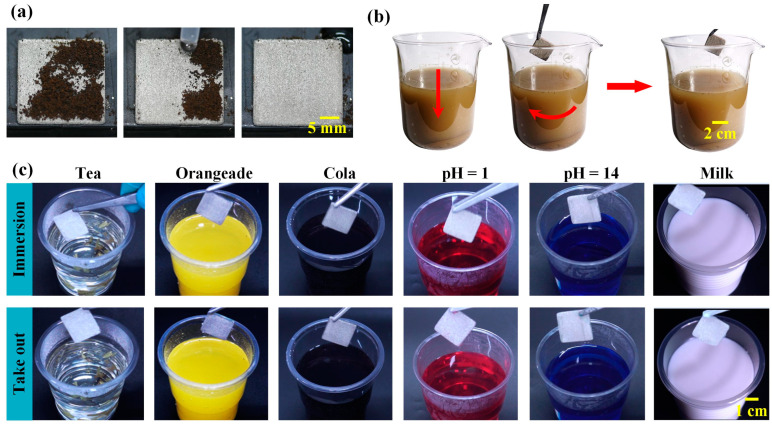
Self-cleaning and antifouling performance. (**a**) Self-cleaning performance of the superhydrophobic nickel plate for solid contaminant (coffee grounds). (**b**) Antifouling performance of the superhydrophobic nickel plate in muddy water environment. (**c**) Antifouling performance of the superhydrophobic nickel plate in various types of liquid contamination environments.

To demonstrate the usefulness of superhydrophobic nickel samples in contaminated environments, a series of tests was conducted [42,43,44]. These included a self-cleaning test, as shown in Figure 5a, and antifouling tests, as shown in Figure 5b,c. In the self-cleaning test, a simulated contaminant (coffee grounds) was intentionally spilled on the superhydrophobic nickel surface. The contaminant was easily carried away and cleaned by the sliding water droplets. Furthermore, in the antifouling test, the superhydrophobic nickel sample was immersed in muddy water and stirred for 10 min; the surface remained clean and was not contaminated by the muddy water. After the antifouling test, we once again measured the contact angle and rolling angle, as shown in Figure S4. It still met the requirements of superhydrophobicity. The superhydrophobic nickel plate surface was also resistant to contamination from a series of liquids, such as acid (pH = 1, HCl solution), alkali (pH = 14, NaOH solution), tea, orangeade, cola, and milk. This experiment aimed to simulate a realistic situation where the surface was exposed to potential fouling agents. Despite the challenging conditions, the superhydrophobic nickel plate remained resistant to fouling, indicating that the superhydrophobic nickel plate surface exhibited good self-cleaning and antifouling properties in various polluted environments. 

**Figure 6:**
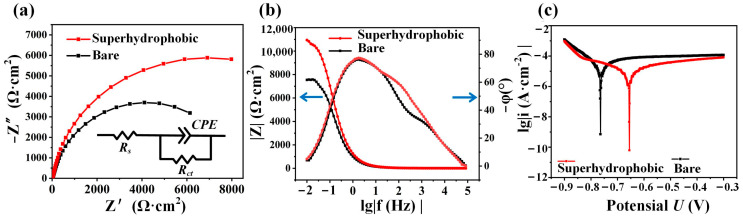
Electrochemical impedance spectroscopy. (**a**) Nyquist plot. (**b**) Bode plot. (**c**) Polarization curve plot.

To quantitatively characterize the corrosion protection of the superhydrophobic nickel sample, additional electrochemical impedance spectroscopy tests were conducted [45,46]. The electrodes were held for 500 s at the open-circuit potential (OCP) before starting the measurements. The results showed that the OCP of the superhydrophobic nickel sample was −68 mV, and the OCP of the bare nickel sample was −222 mV. The EIS parameters were tested at an AC potential of 5 mV, and the applied frequency extended from 10,000 to 0.01 Hz. The Nyquist plot revealed the interfacial charge transfer resistance, as shown in Figure 6a. In general, the larger the arc radius of the Nyquist plot, the higher the charge transfer resistance. The superhydrophobic nickel sample had a larger arc radius compared to the bare nickel sample, which meant that the superhydrophobic nickel sample significantly reduced the charge transfer ability and conductivity of the electrode. Therefore, the superhydrophobic nickel sample enhanced corrosion protection. We used the Randles–Ershler circuit model to fit the equivalent electrical circuit; the values for Rs, Rct, CPE, and Error are shown in Table S1 [47]. Figure 6b shows the Bode plot, wherein the higher low-frequency impedance mode value of the superhydrophobic nickel sample also indicated stronger corrosion protection. 

The results of the polarization curves are shown in Figure 6c. The corrosion potential of the superhydrophobic nickel plate was determined as the potential at the tip of the intersection of the two curves, which was about −655 mV. In comparison, the corrosion potential of the bare nickel sample was about −764 mV. A higher corrosion potential indicated greater corrosion protection of the surface. Remarkably, the corrosion potential of the superhydrophobic nickel sample was 109 mV higher than that of the bare nickel sample. In addition, we conducted fitting calculations for the corrosion rate, Tafel slope, and corrosion current density, and the values are shown in Table S2. Consequently, it can be deduced from the electrochemical impedance spectroscopy tests that the superhydrophobic nickel sample exhibited superior corrosion protection compared to the bare nickel sample.

**Figure 7:**
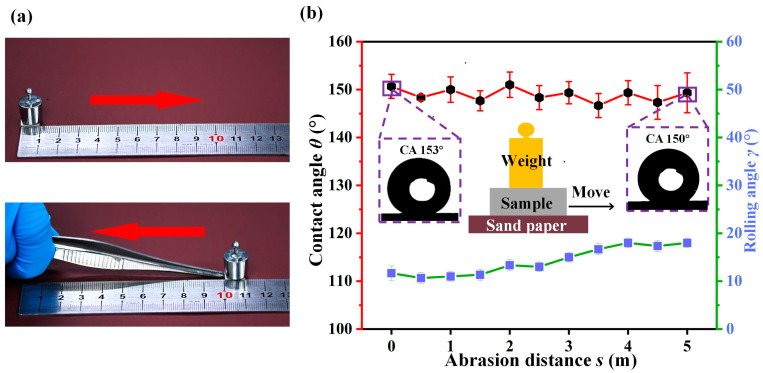
Abrasion resistance test. (**a**) The process of abrasion resistance test. (**b**) Abrasion resistance test data plot and test schematic.

To evaluate the robustness and durability of a superhydrophobic nickel sample in practical applications, we conducted an abrasion resistance test [48,49]. As shown in Figure 7a, we applied a weight onto the nickel plate and used tweezers to rub the superhydrophobic surface against 1200 # sandpaper. We recorded the values of the contact angle and rolling angle while moving on sandpaper. The test schematic is shown in Figure 7b. After 5 m of abrasion, the contact angle was almost unchanged and still met the superhydrophobic requirements, as shown in Figure 7b. Additionally, the water droplets can easily roll off the surface at an inclination angle of 20°. We also used 240 # sandpaper to repeat this test, and the results were similar and shown in Figure S5, indicating that the superhydrophobic nickel sample still maintained good superhydrophobicity after 5 m of abrasion.

## 4. Conclusions

In this work, we proposed a new electrochemical etching method using a wire cathode to fabricate superhydrophobic nickel samples with high current density and high efficiency. Through the establishment of the simulation models of different shapes of cathodes by COMSOL Multiphysics 5.6 software, the results showed that the introduction of a wire cathode greatly improved the current density in the anode region. The processing parameters were investigated, and it was found that the superhydrophobicity was optimal at 30 μm/s feed rate, 30 g/L NaCl electrolyte concentration, and 8 V voltage. The superhydrophobic nickel sample with a contact angle of 153° and a rolling angle of 10° could be fabricated via wire electrochemical etching and modification. The nickel plate was etched with corrosion micro-pits, which reduced the solid-liquid contact area and improved superhydrophobicity. According to XRD and EDS, the element types and composition of the crystalline material of the nickel surface did not change much after wire electrochemical etching. The corrosion protection of the superhydrophobic nickel sample was evaluated in detail by an electrochemical impedance spectroscopy test, and the superhydrophobic nickel sample also had good self-cleaning performance, antifouling performance, and abrasion resistance. The test proved that its corrosion protection exceeded that of a bare nickel sample, which is expected to greatly promote the application of superhydrophobic nickel samples in harsh corrosive conditions.

## Figures and Tables

**Table 1:** Parameters of the wire electrochemical etching and plate electrochemical etching.

	Plate Cathode	Wire Cathode
Anode length	10 mm	10 mm
Cathode length (diameter)	10 mm	100 μm
Gap between the anode and cathode	20 mm	30 μm
Initial potential	8 V	8 V
Anode size	10 mm × 10 mm	10 mm × 10 mm

## Data Availability

Data are contained within the article and supplementary materials.

## Supplementary Materials

The following supporting information can be downloaded at: https://www.mdpi.com/article/10.3390/ma16237472/s1. Figure S1. Plate electrochemical etching simulation. (a) Model for plate electrochemical etching. (b) Local magnification for plate electrochemical etching. (c) Voltage for plate electrochemical etching. (e) Current density for plate electrochemical etching. (f) Current density data on the surface of anode nickel plate. Figure S2. Effect of electrolyte concentration on fabricating superhydrophobic surface. Macroscopic, SEM, and contact angle measurements after etching at 15 g/L, 20 g/L, 25 g/L, 30 g/L, and 35 g/L electrolyte concentration at 30 μm/s feed rate and 8 V voltage. Figure S3. Effect of voltage on fabricating superhydrophobic surface. Macroscopic, SEM, and contact angle measurements after etching at 4 V, 6 V, 8 V, 10 V, and 12 V voltage at 30 g/L electrolyte concentration and 30 μm/s feed rate. Figure S4. Characterization of superhydrophobic nickel surface after antifouling test.(a) Measurement of contact angle.(b) Measurement of rolling angle. Figure S4. Abrasion resistance test data plot on the 240 # sandpaper. Table S1. The fitting values for the electrochemical impedance spectroscopy simulation. Table S2. The fitting values for the electrochemical impedance spectroscopy simulation.
