# Supplementary material for: Wire Electrochemical Etching of Superhydrophobic Nickel Surfaces with Enhanced Corrosion Protection
Source: Materials (Basel). 2023 Dec 1;16(23):7472. doi: 10.3390/ma16237472 (PMC10706957; doi:10.3390/ma16237472)
Supplement: Supplementary file 1 [file materials-16-07472-s001.zip › materials-2710722-supplementary.pdf]

Wire Electrochemical Etching of Superhydrophobic Nickel Surfaces with Enhanced Corrosion Protection

Binghan Wu [1,†], Defeng Yan [1,†], Junyi Lin [1] and Jinlong Song [1,2,*]

[1] State Key Laboratory of High-Performance Precision Manufacturing, Dalian University of Technology, Dalian 116024, China; wubinghan@mail.dlut.edu.cn (B.W.); yandefeng1994@163.com (D.Y.); linjy1372@163.com (J.L.)
[2] Key Laboratory for Micro/Nano Technology and System of Liaoning Province, Dalian University of Technology, Dalian 116024, China
* Correspondence: songjinlong@dlut.edu.cn
† These authors contributed equally to this work.

Figures

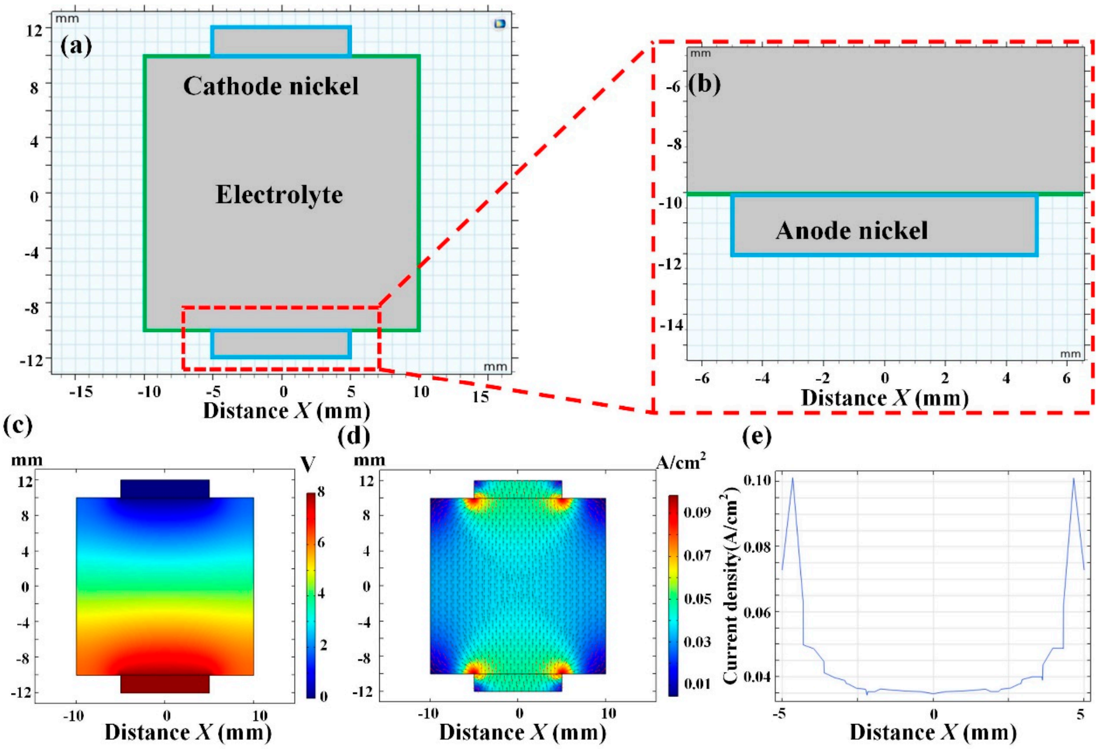

Figure S1. Plate electrochemical etching simulation. (a) Model for plate electrochemical etching. (b) Local magnification for plate electrochemical etching. (c) Voltage for plate electrochemical etching. (e) Current density for plate electrochemical etching. (f) Current density data on the surface of anode nickel plate.

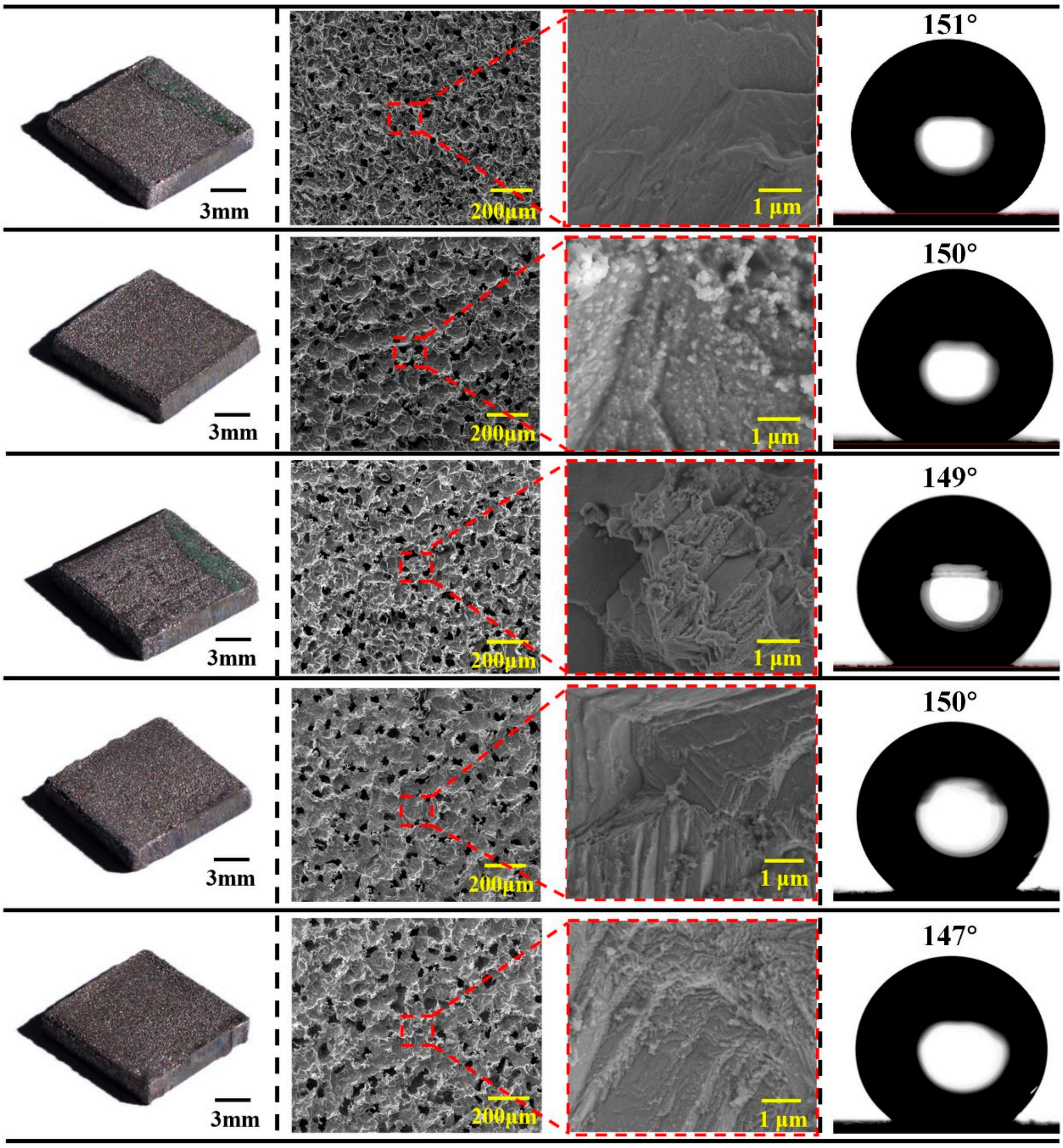

Figure S2. Effect of electrolyte concentration on fabricating superhydrophobic surface. Macroscopic, SEM, and contact angle measurements after etching at 15 g/L, 20 g/L, 25 g/L, 30 g/L, and 35 g/L electrolyte concentration at 30 μm/s feed rate and 8 V voltage.

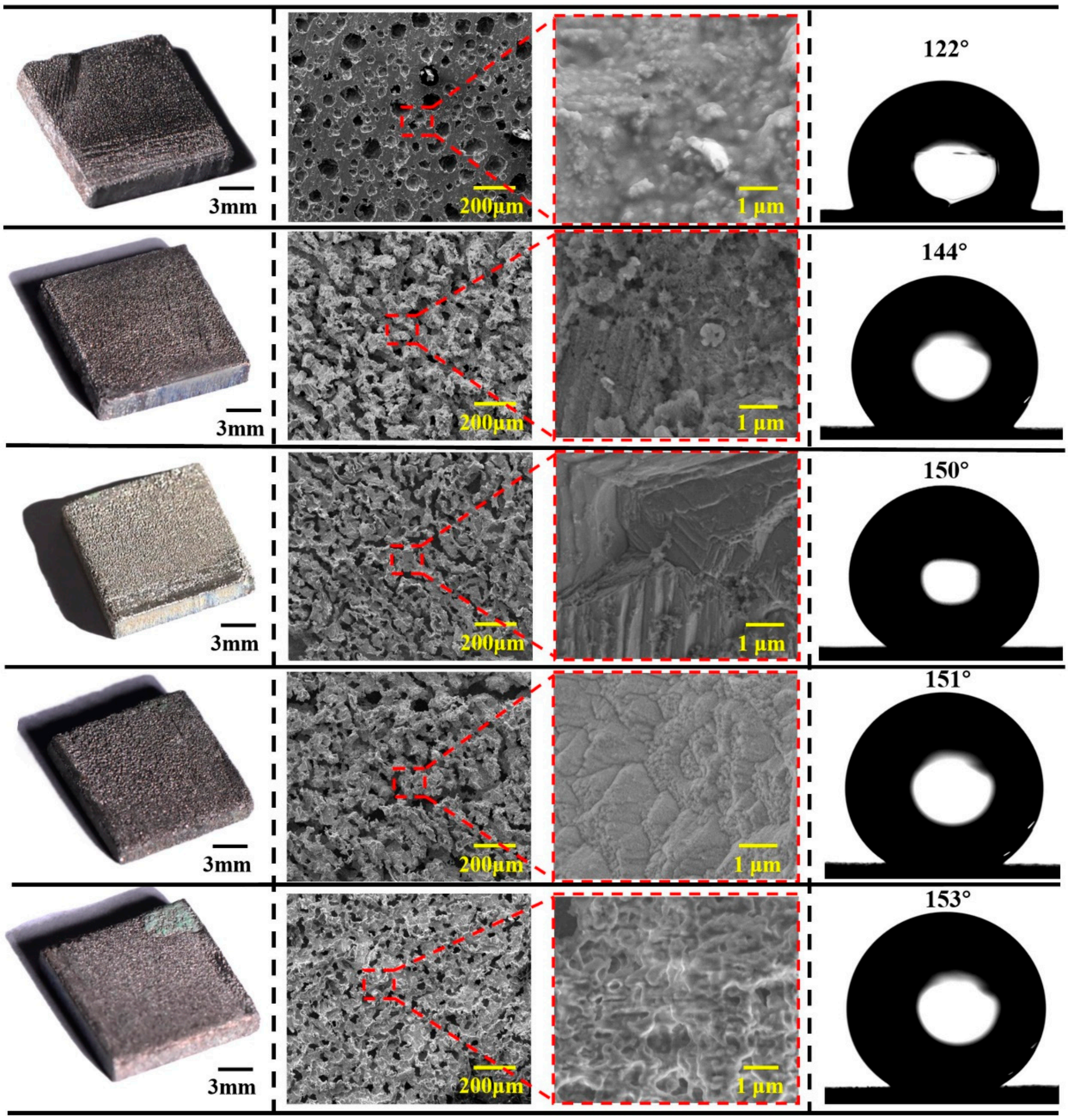

Figure S3. Effect of voltage on fabricating superhydrophobic surface. Macroscopic, SEM, and contact angle measurements after etching at 4 V, 6 V, 8 V, 10 V, and 12 V voltage at 30 g/L electrolyte concentration and 30 μm/s feed rate.

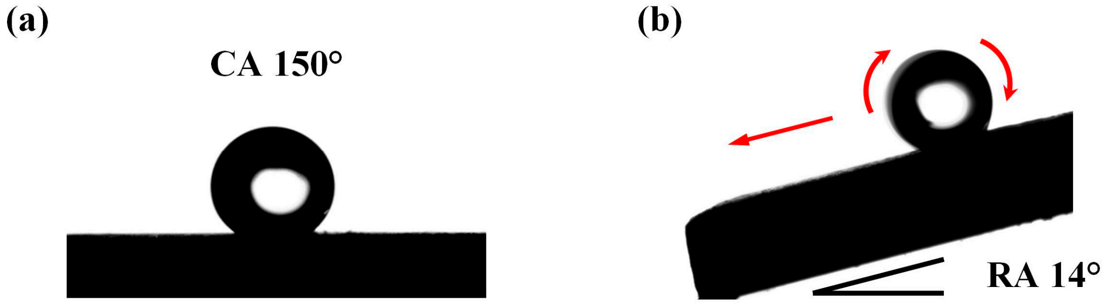

Figure S4. Characterization of superhydrophobic nickel surface after antifouling test.(a) Measurement of contact angle.(b) Measurement of rolling angle.

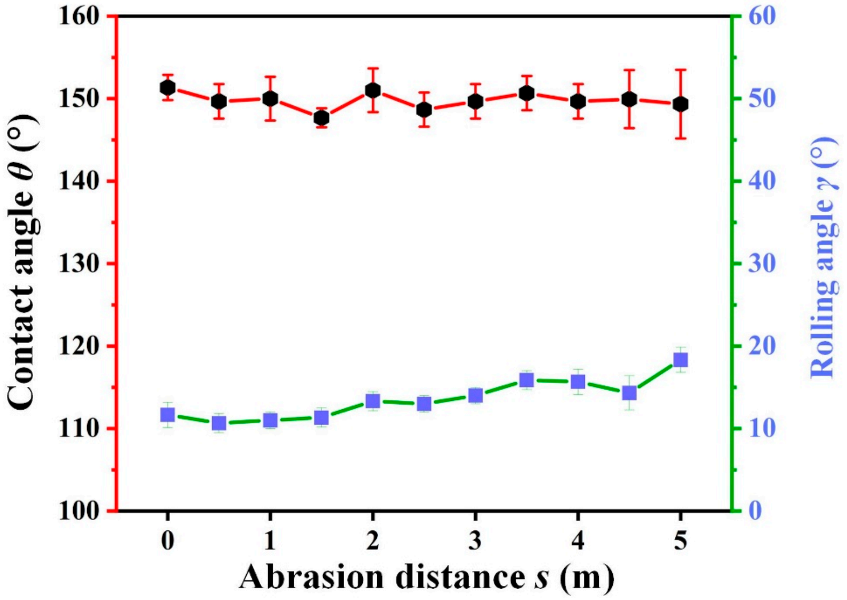

Figure S5. Abrasion resistance test data plot on the 240 # sandpaper.

Tables

Table S1. The fitting values for the electrochemical impedance spectroscopy simulation

Samples	R_s [error(%)]	CPE(μ F) [error(%)]	R_{ct}(Ω) [error(%)]
Superhydrophobic	2.77(5.91)	109.1(2.08)	18952(7.72)
Bare	2.70(4.70)	145.0(2.52)	13069(8.99)

Table S2. The fitting values for the electrochemical impedance spectroscopy simulation

Samples	β_a (V/dec)	$-\beta_c$ (V/dec)	Corrosion rate (mm/a)	corrosion current density(A/cm^2)
Superhydrophobic	86	-115	0.024	$9.77*10^{-6}$
Bare	148	-75	0.051	$3.23*10^{-5}$
